# A New Definition of the Term “High-Phenolic Olive Oil” Based on Large Scale Statistical Data of Greek Olive Oils Analyzed by qNMR

**DOI:** 10.3390/molecules26041115

**Published:** 2021-02-19

**Authors:** Panagiotis Diamantakos, Kostas Ioannidis, Christos Papanikolaou, Annia Tsolakou, Aimilia Rigakou, Eleni Melliou, Prokopios Magiatis

**Affiliations:** 1 Laboratory of Pharmacognosy and Natural Products Chemistry, Department of Pharmacy, National and Kapodistrian University of Athens, Panepistimiopolis Zografou, 15771 Athens, Greece; pdiam@pharm.uoa.gr (P.D.); papanik@pharm.uoa.gr (C.P.); atsolakou@pharm.uoa.gr (A.T.); aimrigakou@pharm.uoa.gr (A.R.); emelliou@pharm.uoa.gr (E.M.); 2 Laboratory of Sylviculture, Forest Genetics and Biotechnology, Institute of Mediterranean and Forest Ecosystems, Hellenic Agricultural Organization “Demeter”, Ilissia, 11528 Athens, Greece; ioko@fria.gr

**Keywords:** olive oil, phenols, NMR, health claim

## Abstract

In the last few years, a new term, “High-phenolic olive oil”, has appeared in scientific literature and in the market. However, there is no available definition of that term regarding the concentration limits of the phenolic ingredients of olive oil. For this purpose, we performed a large-scale screening and statistical evaluation of 5764 olive oil samples from Greece coming from >30 varieties for an eleven-year period with precisely measured phenolic content by qNMR. Although there is a large variation among the different cultivars, the mean concentration of total phenolic content was 483 mg/kg. The maximum concentration recorded in Greece reached 4003 mg/kg. We also observed a statistically significant correlation of the phenolic content with the harvest period and we also identified varieties affording olive oils with higher phenolic content. In addition, we performed a study of phenolic content loss during usual storage and we found an average loss of 46% in 12 months. We propose that the term high-phenolic should be used for olive oils with phenolic content > 500 mg/kg that will be able to retain the health claim limit (250 mg/kg) for at least 12 months after bottling. The term exceptionally high phenolic olive oil should be used for olive oil with phenolic content > 1200 mg/kg (top 5%).

## 1. Introduction

**Figure 1:**
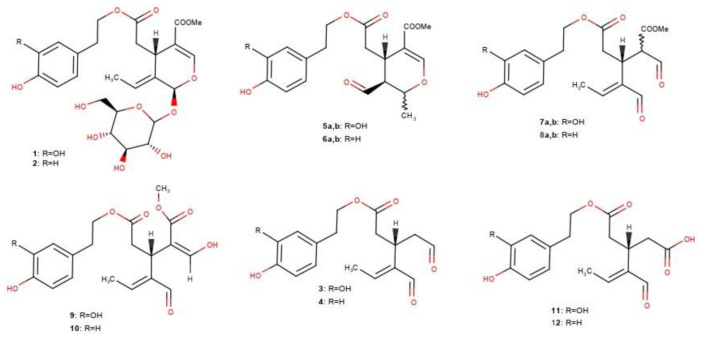
The structures of the studied phenolic compounds.

Olive oil is considered as a healthy fat due to the high content of monounsaturated lipids and the presence of phenolic ingredients with several health preventive bioactivities. Olive oil, apart from having a beneficial lipid composition for human health, is also an excellent source of phenolic substances with excellent health protection properties. European Regulation 432/2012 distinguishes olive oils in terms of their effect on health, depending on their content of these substances [1]. More specifically, it recognizes that olive oil polyphenols contribute to the protection of blood lipids from oxidative stress if it contains at least 5 mg of hydroxy tyrosol and its derivatives (oleuropein and tyrosol complex) per 20 g of olive oil. A variety of compounds (Figure 1) related to oleuropein (**1**) and ligstroside (**2**) like oleacein (**3**) [2], oleocanthal (**4**) [2], oleuropein (**5a**,**b**), and ligstroside aglycons (**6a**,**b**) [3] and their dialdehydic, monoaldehydic and enolic forms (known also as oleuropeindials (**7a**,**b**), ligstrodials (**8a**,**b**), oleomissional (**9**), oleokoronal (**10**) [4] have been recognized as belonging to the class of phenols that should be measured in order to support the above health claim. In addition, oleaceinic acid (**11**) and oleocanthalic acid (**12**) have been recently described and should be included in the above list [5,6].

Oleocanthal is a phenolic compound with anticancer [7,8] and anti-inflammatory activity similar to ibuprofen [9] able to inhibit the progress of Alzheimer’s disease [10,11] as well as oleuropein aglycon [12]. Oleacein presents anti-inflammatory [13] antiatherosclerotic [14] antioxidant [15] and neuroprotective activity [16].

All these polyphenols are found in olive oil in different concentrations, depending, among others, on the harvest season and the oil production conditions. A very important factor that affects the phenolic profile, however, is the variety of olive tree used [17,18]. Designation of an olive oil as “health-protecting food product” can be adopted—when its phenolic content exceeds 250 mg/kg [1], which consequently lead to increased commercial value.

In the last few years, several clinical or in vivo studies have been performed using olive oil with high phenolic content in comparison to moderate or low content to prove the role of the phenolic ingredients in the health-protecting properties of olive oil [19,20,21,22,23,24,25,26,27]. As a consequence, a new term, “High-phenolic olive oil”, has appeared in scientific literature and in parallel in the market. However, there is no available definition of that term regarding the concentration limits of the phenolic ingredients of olive oil.

Some previous studies have attempted to measure the concentration range of the phenolic ingredients of olive oil but they are based on results from a relatively small number of samples and a short monitoring period. In addition, the phenolic content has been measured with an array of different methods (colorimetric, chromatographic or spectroscopic) and the results are expressed in different units creating difficulties for comparison. Another factor making the comparison even more complex is the use of olive oil samples either produced from industrial olive mills or under laboratory conditions. For example, in the early studies of olive oil phenolic content, it had been reported that the range was between 50 and 1000 mg/Kg caffeic acid equivalents with usual values between 100 and 300 mg/kg, measured by Folin-Ciocalteu [28]. Similarly, in an early study of 79 samples of cv. Hojiblanca and cv. Picual from Spain, the range of the total phenol content was 20−457 mg/Kg caffeic acid equivalents [29] while a later study of 134 samples from Greece collected during one year showed a range 60–512 mg/kg gallic acid equivalents [30]. In another study of Greek varieties with a higher sample pool (221 samples) the range of total phenols was 23–641 mg/kg and the mean value was 151 mg/k,g measured by ^31^P-NMR [12]. In a more recent study of olive oils from Greek varieties, in a pool of 340 industrial samples, the range was found to be 20–1530 mg/Kg measured by ^1^H-NMR [3] while another study of laboratory produced samples from 36 different varieties using HPLC-UV, reported similar ranges (26–1410 mg/Kg) [31]. It should be noted that other qNMR methods for measurement of the phenolic ingredients of olive oil have also been recently developed [32] reporting total phenolic content up to 851 mg/kg from 32 Italian samples. Recently, a study by LCMS on laboratory produced olive oils reported minimum and maximum values of individual phenolic compounds in a study of 160 samples from 80 cultivars [33] and the most recent study of 44 varieties monitored for three years with the same methodology showed a diverse total phenolic content ranging up to 4497 mg/Kg [34].

All the above-described approaches show us that to define the term “high-phenolic olive oil”, it is necessary to obtain results from a large number of industrially produced samples recorded during a long period of several years and analyzed by the same methodology. For this purpose, we performed a statistical evaluation of the phenolic content of the olive oil samples that have been recorded since 2009 in the olive oil database of the Laboratory of Pharmacognosy of the National and Kapodistrian University of Athens, that was significantly enriched by the Interreg-Med “ARISTOIL” project during 2016–2020.

The phenolic content of the olive oil samples has been measured using a reliable analytical method based on 1D qNMR spectroscopy that was first published in 2012 [2,3,4] and then expanded to measure the concentration of all the compounds mentioned in the regulation with a simple experiment, avoiding the formation of artifacts and providing the necessary data for the certification of the health claim [35]. During the period 2009–2020, 7854 samples from 15 countries have been analyzed with this method.

In the current study, we present the results of the statistical evaluation of the samples originating from Greece (*n* = 5764), coming from more than 30 varieties, and recorded for an eleven-year period concerning their phenolic content and their relationship with harvest period and variety. To date, the current work is the largest statistical study of the phenolic content of olive oil and aims to be a reference study for the future. In addition, we present some new results coming from a previous study that had been partially published [5] regarding the loss of phenolic content during storage. 

The results of the statistical evaluation in combination with the results of the storage study were used to provide a first definition of the term “high phenolic olive oil”.

## 2. Results and Discussion

### 2.1. Overall Analysis

Table 1 shows the mean and the maximum concentrations of the studied phenolic ingredients for the Greek analyzed samples. The term oleuropein and ligstroside aglycon correspond to the monoaldehydic closed ring forms **5a**,**b** and **6a**,**b** and the term dialdehyde oleuropein and ligstroside aglycon correspond to the open ring dialdehydic forms **7a**,**b** and **8a**,**b** which are in equilibrium with the monoaldehydic enolic forms **9** and **10**, respectively. The term total tyrosol derivatives corresponds to the sum of oleocanthal and all the ligstroside aglycons while the term total hydroxytyrosol derivatives corresponds to the sum of oleacein with all the oleuropein aglycons. The term total phenols corresponds to the sum of total tyrosol and total hydroxytyrosol derivatives. All the olive oil samples have been analyzed shortly after their production and for this reason phenolic products related to hydrolysis (e.g., free tyrosol or hydroxytyrosol) or oxidation (e.g., oleocanthalic acid or oleaceinic acid) had very low concentrations (<10 mg/kg) not significantly affecting the total phenolic content and were not evaluated statistically.

Although the current study is focused only on the samples of Greek origin, it is noteworthy that among all the 7854 samples from 15 countries, the highest total phenolic content (4947 mg/kg) was recorded from a sample from Cyprus (Atsas) from Kalamon variety with oleocanthal content 3762 mg/kg produced in September 2017. To date, these two values of total phenols and oleocanthal are the highest recorded in comparison to all available literature data. 

In previous studies [2,3] we had presented some initial statistical data, but the number of the analyzed samples was much smaller (*n* = 340). The maximum concentrations of oleocanthal (711 mg/kg) or total phenols (1534 mg/kg) were lower than those presented herein, reflecting the effort of several producers during the last few years to follow practices that increase the phenolic content of their olive oils. 

Small-scale statistical data have also been described from other studies [29,30,31,32,33,34] but as mentioned in the introduction, the comparison among them presents several problems due to the different methodologies used for the production and the analysis of the olive oil samples. For example, in a previous study, 134 samples from Greece measured by HPLC-DAD showed oleocanthal ranging from 0 to 512 mg/kg [30], while a study from 32 Italian samples by HPLC and NMR showed oleocanthal up to 266 mg/kg and a total up to 830 mg/kg [32]; a study of 80 varieties showed oleocanthal up to 2931 mg/kg [33] and another one with 44 varieties showed total phenolic content up to 4497 mg/kg [34].

**Figure 2:**
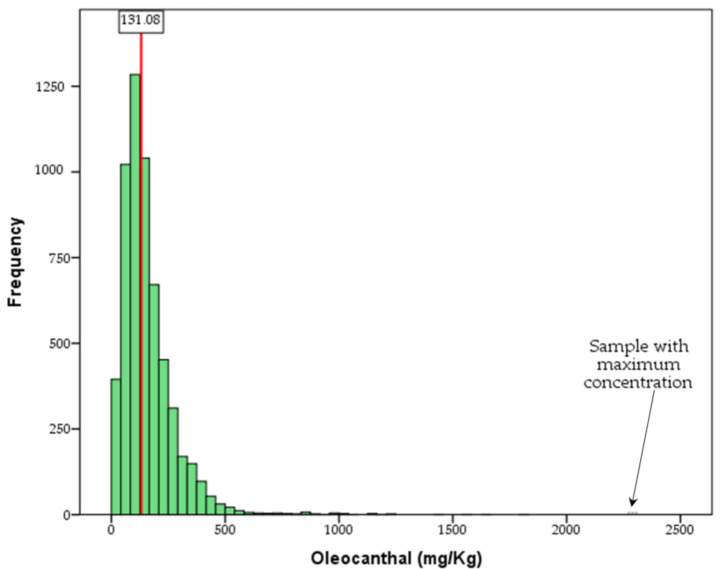
The oleocanthal distribution of the analyzed olive oil samples (*n* = 5764). The red line represents the median.

**Figure 3:**
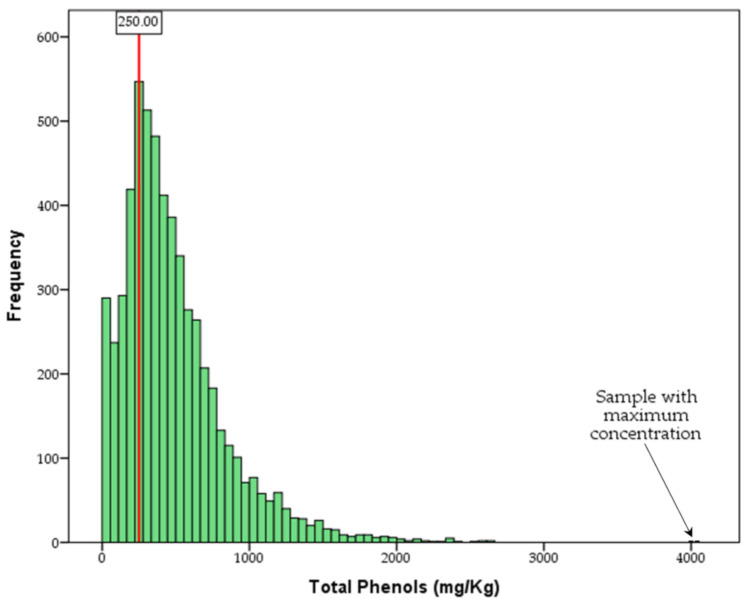
The total phenol content distribution of the analyzed olive oil samples (*n* = 5764). The red line represents the EU health claim limit).

**Figure 4:**
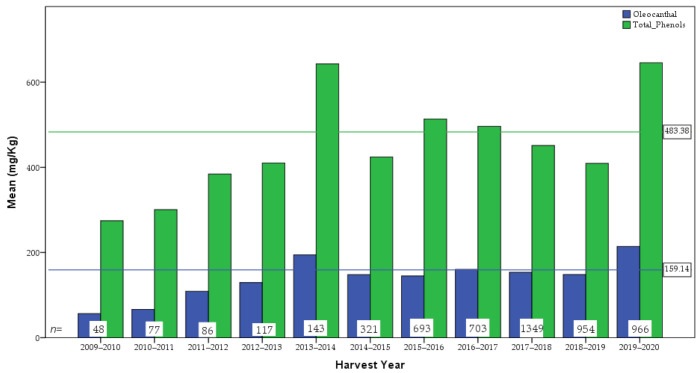
Difference in concentration of oleocanthal and total phenols in relation to harvest year (the horizontal green and blue lines represent the overall mean of oleocanthal and total phenols, respectively. The number in the base of the bars represents the sample size of each year).

Figure 2 and Figure 3 show the oleocanthal and total phenol content distribution of the analyzed olive oil samples, while Figure 4 shows the difference in concentration of oleocanthal and total phenols in relation to harvest year. In the current study, special attention has been given to oleocanthal since this is a compound that has attracted the interest of the scientific community and the market.

From Figure 2, it is obvious that very few samples present oleocanthal content higher than 500 mg/kg, although there are samples with oleocanthal content higher than 1000 or even 2000 mg/kg. The reason behind this large variability is most probably genetic. As explained in Section 2.3, specific varieties like Kalamon or Lianolia Kerkyras have an increased tendency to produce high amount of oleocanthal; however, the impact of other factors (e.g., pedoclimatic) is still under investigation. 

Concerning the total phenols distribution (Figure 3), again, the large variability is visible, but it is very important to note that the majority of the analyzed samples (74%, also see Table S1 in Supplementary Materials) presents a phenolic content higher than the EU health claim limit of 250 mg/kg (or 5 mg/20 g). This observation, based on a long-term recording and for a large number of samples, proves that the Greek olive oil production on average fulfils international requirements of olive oil with certified health claim referred to the phenolic content. The distribution of the rest of the studied phenols are presented in Supplementary Materials (Figures S1–S8).

Regarding the annual variation of the total phenolic content, two possible factors should be noted. The first one is that after 2012–2013, when the EU health claim was established, many producers showed higher interest for the phenolic content and started to follow practices to increase it (e.g., early harvest). The second one is related with the impact of phytopathological factors. More specifically, in years like 2018–2019, Greek production suffered from extended damage from the olive fly (*Bactrocera oleae*) and the olive anthracnose caused by *Colletotrichum* spp. that negatively affected the quality and also the phenolic content of olive oil.

### 2.2. Differences among Months of Harvest

**Figure 5:**
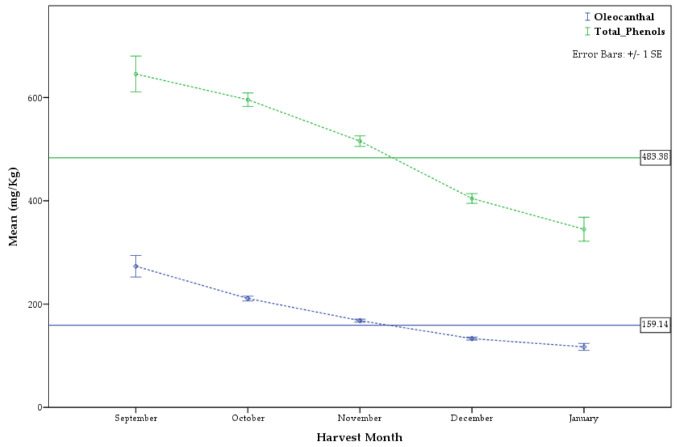
Difference in concentration of oleocanthal and total phenols in relation to harvest month.

**Figure 6:**
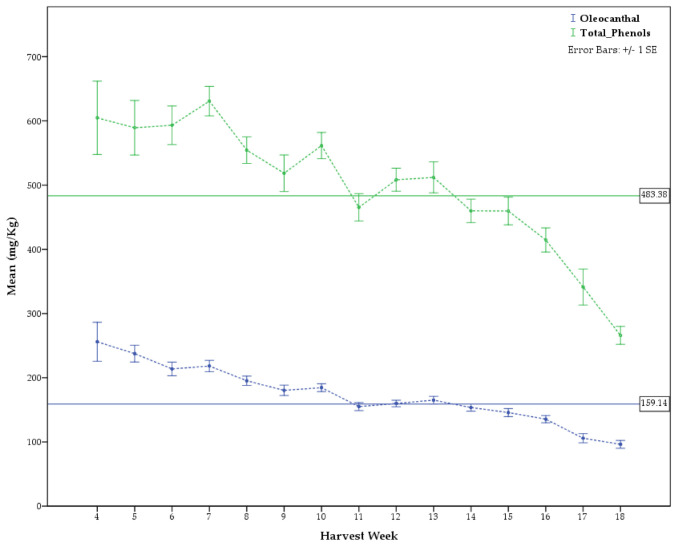
Difference in concentration of oleocanthal and total phenols in relation to harvest week.

As many producers share the common practice to mix olive oil produced during several months, many analyzed samples could not be used to correlate the phenolic content with the harvesting week or month. Among the total 5764 samples, 3440 were certainly produced during specific time-ranges and, therefore, used to study the impact of harvest time on the phenolic content. All the results of the post hoc tests for the multiple comparisons, using Duncan’s Multiple Range Test (MRT), are presented in Table 2 and Table 3. Figure 5 and Figure 6 present the phenol concentrations in relation to harvest month and harvest week, respectively.

We have recently studied [35] the effect of harvest time monitoring specific trees for a whole period (from September to February) and we were able to observe a decreasing trend. Many researchers have also reported that the concentration of phenols in olive oil decreases during ripening [36,37,38,39,40] while others present a nonlinear relation between olive ripeness and phenol concentration and especially, total phenolic content seems to increase in the early stages reaching a maximum concentration and after this point there is a decrease during ripening [41,42].

Table 2 as well as Figure 5 and Figure 6 show clearly that independently of the variety, the geographic origin, the type of olive mill or the climatic conditions every year, there is a decreasing trend of the phenolic content from September to January (Supplementary Materials Figures S9–S11). The same trend can also be observed for oleocanthal, which is considered a very important ingredient of olive oil and many producers seek high oleocanthal content.

The phenols concentrations differed statistically significantly (all studied substances at *p* ≤ 0.001 except ligstroside aglycon at *p* ≤ 0.05) among harvest month, indicating that harvest month affects the olive oil phenol content. September was the month in which the produced oils displayed the highest averaged content of oleocanthal (Table 2), sum of oleocanthal and oleacein (Table S2), ligstroside aglycon (Table S3), total tyrosol derivatives (Table S4) and total phenols (Table 2). October was recorded instead as the month with the maximum production of oleacein (Table S5), oleuropein aglycon (Table S6), dialdehyde ligstroside aglycon (Table S7), dialdehyde oleuropein aglycon (Table S8) and total hydroxytyrosol derivatives (Table S9). September production was ranked second in the relative content of oleacein, dialdehyde ligstroside aglycon and total hydroxy tyrosol derivatives, while November for oleuropein aglycon and dialdehyde oleuropein aglycon. However, there was no statistically significant difference in the mean phenolic concentrations between September and the respective top ranked months in relation to the above substances except oleocanthal, sum of oleocanthal and oleacein and total tyrosol derivatives (Table 2, Tables S3 and S8, respectively).

### 2.3. Differences among Varieties

It is well known that the phenolic profile of the olive oil produced by each olive variety presents significant variability [33,34]. Some varieties are mainly producing decarboxylated secoiridoid derivatives like oleocanthal and oleacein, others are mainly producing oleuropein and ligstroside aglycons and others can produce increased amounts of flavonoids and lignans. In addition, some varieties have inherited tendency to produce increased amounts of specific phenolic ingredients in the corresponding olive oil [35], a characteristic which is related with the activity of specific enzymes and is genetically regulated. The latter characteristic is empirically well known to the organoleptic panel tasters who discriminate the varieties according to their taste as intense bitter, pungent or mild

In Greece, there are more than 100 recorded varieties but only a small number of them is widely cultivated. It is estimated that about 70% of the Greek production comes from the Koroneiki variety. In our study, 53% of the samples were belonging to the Koroneiki variety (*n* = 2649) and the remaining to 11 cultivated varieties or to feral (wild) olive trees. The total number of samples that were included in the statistical analysis was 4995. The remaining samples until the total number of 5764 was either belonging to cultivars with less than 30 samples or to samples coming from mixed orchards.

All the subsets from the post-hoc multiple comparisons for the chemical studied traits are presented in Table 3, Table 4 and Tables S10–S17 in Supplementary Materials. Kalamon and Lianolia Kerkyras varieties showed the highest mean concentration of oleocanthal and oleacein, respectively (Table 3, Tables S10 and S11), with statistically significant difference with the rest of the varieties. It should be emphasized that the maximum value of oleocanthal 2272 mg/kg reported in Table 1 has been obtained from an olive oil from Kalamon variety. Extremely high oleocanthal content has also been recorded from the same variety grown in Spain (2776 mg/kg) [33] confirming the genetically defined capability of this variety to produce high amounts of oleocanthal. Kalamon and Lianolia together with Zakynthou which ranked third and second, respectively, and with Olympia, which ranked fourth and third, formed a group with the highest concentrations for oleocanthal and oleacein (Table 3 and Table S10). The varieties of Agrielia (wild), Chalkidikis and Athinolia were consisting a second group, with oleocanthal and oleacein above the average (Table 3 and Table S10).

Olympia variety was the outstanding variety in the concentration of oleuropein aglycon, ligstroside aglycon, dialdehyde ligstroside aglycon, dialdehyde oleuropein aglycon (Tables S12–S15) and total hydroxy tyrosol derivatives (Table S16). Kalamon was the outstanding variety in the concentration of total tyrosol derivatives (Table S17). The varieties Athinolia, Lianolia, Kalamon and Chalkidikis were classified in the following places depending to the substance being measured (Table 3, Table 4 and Tables S10–S17).

Concerning the total phenolic content, the variety with highest mean value was Olympia, followed by the varieties Zakynthou, Kalamon and Lianolia Kerkyras (Table 4). It should be noted the highest total phenolic content 4003 mg/kg reported in Table 1 has been obtained from Kalamon variety. It is also interesting to note that although Koroneiki variety showed a mean value a little lower than the general mean, it also showed a big variability ranging from 0 to 2637 mg/kg. This finding highlights the role of factors such as the olive mill conditions or the pedoclimatic influence. 

**Figure 7:**
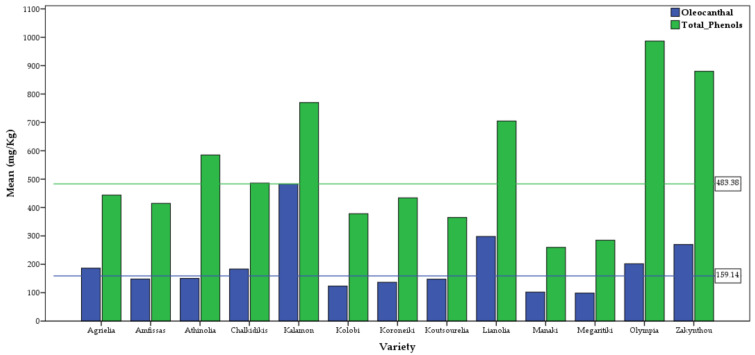
Difference in concentration of oleocanthal and total phenols in relation to variety (the horizontal green and blue lines represent the overall mean of oleocanthal and total phenols, respectively).

The relationship between oleocanthal and total phenols in each variety is presented in Figure 7. It is obvious that in most Greek varieties oleocanthal is an important fraction of the total phenol content, especially for Kalamon (61%) and Lianolia (40%). In contrast, in varieties like Olympia (17%) oleocanthal is a smaller fraction because the aglycons predominate. 

### 2.4. Impact of Other Factors

The statistical evaluation of the chemical analysis results has also included other factors like the geographic origin of each sample at the level of region and municipality and also the type of olive mill used. The results are only informative and not leading to a definite conclusion and for this reason they are presented in Supplementary Materials (Tables S18–S20).

### 2.5. Impact of Storage

The results regarding the phenolic concentration, showed that the samples stored in room temperature had an average preservation of 54.1% (±9.46%) of their original total phenolic content after a 12-month storage. When lower temperatures, +4 °C and −18 °C were used for long-term storage (6 months), the samples preserved 92% (±7.35%) to 97% (±2.65%) total phenolic content, respectively (Table S21). Moreover, samples that were stored in the freezer (−18 °C), showed small-scale changes in the phenolic profile, suggesting/indicating it as the best preservation treatment. Their average phenolic content decrease was only 9.50% (±3.08%) after 12 month-storage.

From the point of view of a consumer that is interested in buying an olive oil with a health claim, it is important to know that during usual home storage, the olive oil will lose a percentage of its phenolic content, so the olive oil at bottling time should have at least double concentration than the EU limit of 250 mg/kg. Otherwise, the olive oil should be stored at lower temperature to retain the phenolic content over the limit.

### 2.6. Redefining the Olive Oil Classification According to Its Phenolic Content

In order to rationalize olive oil health claims and to adjust its added commercial value to its total phenol content, the useful tools in quality management and grading of percentiles were used to outline the relative placement of data samples according to their phenol concentration. We examined the placement of the samples constructing a percentile table in 5% and 2% intervals, i.e. using cut points for 20 and 50 equal groups, respectively (Table 5 and Table S1). Since olive oil nutrition and health claims are determined on whether its phenolic content exceeds 250 mg/kg, this study showed that although 74% of all examined samples had total phenols more than 251 mg/kg (Table S1), they were automatically included in a single category despite their total phenol content. Only 26% of the remained samples had lower phenolic content. Moreover, 50% of the samples had phenolic content almost higher than 400 mg/kg and about 25% of the examined olive oil samples had total phenols more than 625 mg/kg. 

About 38% and 17% of the samples had phenol concentration two and three times higher, respectively, than olive oil bearing to nutrition and health claims. In addition, 8% of the samples had phenolic content more than 1000 mg/kg and about 5% of high-quality samples contained phenols over 1180 mg/kg. 

### 2.7. Definition of Limits for High Phenolic Olive Oil

Percentiles and quantiles are statistics used for summarizing the relative location of data within a set according to their magnitude and independently from a particular underlying probability distribution [43,44]. Percentiles are useful tools in the field of quality management to show the distribution of observed performance data and for attributing quality grading and goals in extra-analytical processes through indicators [45,46]. According to the above analysis, the majority of Greek olive oil production had high total phenolic content and was not adequately distinguished from the rest of the production that simply adhere to health claim limits mentioned above. Furthermore, a significant part of olive oil production had extremely high phenolic content, a feature that offers them a particular added value. In order to promote fairness in the olive oil market and proportional equality in olive oil prices, due to the substantial variation in the phenols content, a requirement arises for restructuring the values of total phenol concentrations bearing health claim.

Based on the statistical evaluation we found that the mean value of total phenolic content is 483 mg/kg, which for simplicity could be rounded up to 500 mg/kg. In addition, we observed that during storage in a closed bottle at room temperature, the phenolic content will present a mean decrease of 46% in 12 months. Combining these two measurements we propose that the term high-phenolic should be used for olive oils with phenolic content over 500 mg/kg that will be able to safely retain the health claim limit (250 mg/kg) for at least 12 months after bottling. An additional discrimination would be the term “exceptionally high phenolic olive oil” that should be used only for olive oils with phenolic content over 1200 mg/kg, which corresponds to the top 5%. The above definitions are consumer-oriented and could be used for informative purposes in the market but could also be used in the scientific literature when the scientists want to discriminate a high phenolic from a low phenolic olive oil especially when it is used in clinical or experimental trials. To our knowledge, this is the first attempt to define the term high phenolic olive oil based on robust statistical data. Although the data come mainly from one country, the fact that Greece is the third most important producer country and especially a country with one of the highest percentages of extra virgin olive oil in the world, it increases the validity of the current proposal. Further comparative studies with similar amount of data from other countries would be useful for a commonly accepted definition.

## 3. Materials and Methods

### 3.1. Chemicals and Standards

All solvents were of analytical grade and purchased from Merck. Syringaldehyde (98% purity) used as internal standard (IS) was purchased from Sigma-Aldrich (Steinheim, Germany). IS solution was prepared in acetonitrile at a concentration of 0.5 mg/mL and kept in refrigerator. Prior to use the IS solution was left to come to room temperature.

### 3.2. Instrumentation

The quantitative determination of phenols in olive oil was performed using NMR spectroscopy on a DRX 400 MHz (Bruker). CDCl_3_ was used as a solvent due to its advantage not to react with the studied compound. The spectra were processed using the either the MNova (Mestrelab Research) or the TOPSPIN program.

### 3.3. Olive Oil Samples Origin

The EVOO samples used in the current study were obtained from olives (*Olea europaea* L.) harvested and extracted in 11 consecutive years: November 2009 to September 2020. In total, 7874 samples were obtained from Greece, Italy, Spain, Croatia, Cyprus, Portugal, Turkey, Lebanon, Syria, Egypt, Morocco, USA, Tunisia, Argentina and Chile. The current statistical analysis is related only to olive oil samples originating from Greece (*n* = 5764). In total, samples of more than 30 different varieties were included in the study. From the analysis were eliminated varieties numbered fewer than 30 samples and the statistical analysis was restricted to 13 major varieties: Amfissas (or Piliou or Conservolea), Athinolia (or Mastoides or Tsounati), Chalkidikis, Kalamon (or Kalamata), Kolovi, Koroneiki, Koutsourelia (or Patrinia), Lianolia Kerkyras (or Corfu), Manaki, Megaritiki, Olympia (or Nemoutiana or Atsiholi), Zakynthou, and Agrielia (or wild). The olive oil samples were harvested during August to March every year and were sent for analysis from September to April. The samples that were harvested on August, February and March were eliminated from the analysis due to their limited number. To assess the impact of harvest week, we considered the first week of September the first harvest week. The olive oil production was performed in either two-phase or three-phase mills. All samples were provided by small-scale producers that could guarantee their origin.

### 3.4. Olive Oil Extraction for Analysis

Olive oil (5.0 g) was mixed with cyclohexane (20 mL) and acetonitrile (25 mL). The mixture was homogenized using a vortex mixer (VXMTAL multi-tube vortex mixer, OHAUS) for 30 s and centrifuged at 4000 rpm for 5 min. A part of the acetonitrile phase (25 mL) was collected, mixed with 1.0 mL of a syringaldehyde solution (0.5 mg/mL) in acetonitrile, and evaporated under vacuum using a rotary evaporator (Buchi, Flawil, Switzerland).

### 3.5. NMR Spectra Analysis

The residue of the above procedure was dissolved in CDCl_3_ (750 μL) and an accurately measured volume of the solution (550 μL) was transferred to a 5 mm NMR tube (Norell, Morganton, NC, USA). ^1^H NMR (Bruker DRX400, Billerica, MA, USA) spectra were recorded at 400 MHz. Typically, 32 scans were collected into 32K data points over a spectral width of 0−16 ppm with a relaxation delay of 1 s and an acquisition time of 1.7 s. Prior to Fourier transformation (FT), an exponential weighting factor corresponding to a line broadening of 0.3 Hz was applied. The spectrum was phase corrected and accurate integration was performed manually for the peaks of interest. The quantitation was performed as recently described [35].

### 3.6. Phenolic Content Loss during Storage

For this part of the study, we used a previously described [5] set of 29 EVOO samples of different total phenolic concentrations, which were monitored periodically, regarding their phenolic content, for a period of up to 2 years. The samples were stored in dark glass bottles, with 5% headspace. The bottles were placed either inside a dark cabinet, with an average temperature of 25 °C, or in a refrigerator at 4 or −18 °C, and three replicates of each sample were analyzed every 3 or 6 months.

### 3.7. Statistical Analysis

All the olive oil samples were analyzed to determine the chemical profile of the following target phenolic substances: oleocanthal, oleacein, oleuropein aglycon, ligstroside aglycon, dialdehyde ligstroside aglycon, dialdehyde oleuropein aglycon, total tyrosol derivatives, total hydroxy tyrosol derivatives and total phenols. The chemical profile analysis was studied concerning the impact of harvest time (year, month, week), variety and geographic origin (region, county and municipality) of the samples.

In the data, analysis of variance was applied to control the significance of differences among varieties, harvest year, month, and week as well as in geographical origin of the samples. Duncan’s Multiple Range Test (MRT) was used for the multiple comparisons. The sample size in every analysis of variance was not the same due to outliers’ exclusion from the data. All analyses were performed at a significance level of *p* = 0.05, using SPSS v.20 software for Windows (IBM SPSS Statistics 2011, IBM Corp., Armonk, NY, USA).

## 4. Conclusions

We propose that the term “high-phenolic” should be used for olive oils with phenolic content > 500 mg/kg. Using the measured average loss rate, we conclude that an olive oil with phenolic content > 500 mg/kg will retain the health claim limit (250 mg/kg) for at least 12 months after bottling. The term exceptionally high phenolic olive oil should be used for olive oil with phenolic content > 1200 mg/kg (top 5%).

## Figures and Tables

**Table 1:** The maximum and mean concentrations (mg/kg) of the studied substances for the Greek analyzed samples (*n* = 5764).

Substance	*Maximum*	*Mean*	*Std. Deviation*
Oleocanthal	2275	159	124
Oleacein	1046	94	81
Oleocanthal + Oleacein (D1 index)	3322	244	191
Oleuropein Aglycon	618	40	43
Ligstroside Aglycon	530	36	36
Dialdehyde Ligstroside Aglycon	1559	93	115
Dialdehyde Oleuropein Aglycon	867	56	82
Total Tyrosol Derivatives	2745	286	209
Total Hydroxy Tyrosol Derivatives	1519	189	165
Total Phenols	4003	483	357

**Table 2:** Comparisons of oleocanthal means (mg/kg) and total phenols means (mg/kg) among harvest months using Duncan’s multiple range test (*a* = 0.05) (harvest months reside in the same subset are not statistically different).

Harvest Month	*n*	Subset for Oleocanthal				Subset for Total Phenols		
		1	2	3	4	1	2	3
September	163	273.25				645.58		
October	948		210.85			595.81		
November	1327			168.02			515.53	
December	922				133.12			404.51
January	80				117.13			344.92

**Table 3:** Comparisons of oleocanthal means (mg/kg) for each variety using Duncan’s multiple range test (*a* = 0.05) (varieties reside in the same subset are not statistically different).

Variety	*n*	Subset					
		1	2	3	4	5	6
Kalamon	59	482.6					
Lianolia Kerkyras	350		297.9				
Zakynthou	44			269.6			
Olympia	260				202.0		
Agrielia (wild)	164				186.5		
Chalkidikis	362				183.5		
Athinolia	260					150.3	
Amfissas	276					148.3	
Koutsourelia	189					147.7	
Koroneiki	2649					136.8	
Kolovi	40					123.2	123.2
Manaki	261						102.1
Megaritiki	81						98.4

**Table 4:** Comparisons of Total Phenols means (mg/kg) for each variety using Duncan’s multiple range test (*a* = 0.05) (varieties reside in the same subset are not statistically different).

Variety	*n*	Subset							
		1	2	3	4	5	6	7	8
Olympia	260	986.4							
Zakynthou	44		880.0						
Kalamon	59			769.9					
Lianolia Kerkyras	350			704.9					
Athinolia	260				585.2				
Chalkidikis	362					486.1			
Agrielia (wild)	164					444.0	444.0		
Koroneiki	2649					434.3	434.3		
Amfissas	276					414.7	414.7		
Kolovi	40						378.3		
Koutsourelia	189						364.8	364.8	
Megaritiki	81							285.0	285.0
Manaki	261								259.6

**Table 5:** Percentiles (5%) of phenolic content of the examined samples (*n* = 5764).

Percentiles (%)	Oleocanthal	Oleacein	Sum Oleocanthal Oleacein	Oleuropein Aglycon	Ligstroside Aglycon	Dialdehyde Ligstroside Aglycon	Dialdehyde Oleuropein Aglycon	Total Tyrosol Derivatives	Total Hydroxy Tyrosol Derivatives	Total Phenols
5	34.00	0.00	19.00	0.00	0.00	0.00	0.00	39.54	0.00	54.44
10	52.00	7.97	51.04	0.00	0.00	0.00	0.00	73.89	22.58	119.58
15	64.26	29.64	79.09	11.50	11.28	0.00	0.00	101.62	46.73	174.04
20	74.15	36.48	101.96	14.31	13.61	0.00	0.00	125.70	63.30	212.13
25	84.03	41.61	120.34	17.00	16.75	0.00	0.00	145.76	77.29	244.21
30	93.92	48.44	135.86	20.59	19.18	13.61	0.00	164.46	90.99	271.87
35	102.16	53.57	151.97	22.92	21.62	32.22	0.00	182.04	106.28	303.92
40	112.04	60.21	167.63	25.24	22.92	41.53	14.31	202.19	119.63	334.05
45	120.28	65.53	184.37	28.92	25.24	50.84	24.05	221.66	134.87	365.77
50	131.08	73.39	201.80	31.36	28.01	60.15	28.92	242.53	150.96	402.23
55	141.70	80.92	219.69	33.79	30.22	71.78	36.23	264.23	167.97	441.17
60	153.24	88.00	239.47	36.23	33.79	85.75	43.53	288.33	186.53	482.27
65	168.07	98.76	261.10	41.10	36.88	97.38	53.27	313.32	206.24	526.88
70	182.89	109.97	287.15	44.00	41.10	114.76	63.01	341.99	229.00	576.63
75	202.20	123.64	318.44	50.48	45.97	132.29	77.30	376.61	255.94	638.81
80	224.09	140.73	357.45	55.71	50.84	153.23	92.23	415.67	286.02	706.22
85	253.02	162.95	411.26	65.45	57.82	183.48	116.58	470.92	327.67	796.62
90	294.94	200.55	484.81	80.06	69.46	227.69	153.11	546.19	390.71	933.94
95	367.44	254.45	601.77	109.01	94.18	323.10	223.72	667.57	516.10	1189.71

## Data Availability

The data presented in this study concerning the chemical analysis and the name of the producer of each olive oil sample are available on request from the corresponding author.

## Supplementary Materials

The following are available online, Table S1. Percentiles (2%) of phenolic content of the examined samples (*n* = 5764). Table S2. Comparisons of sum oleocanthal oleacein means (mg/kg) among harvest months using Duncan’s multiple range test (*a* = 0.05) (harvest months reside in the same subset are not statistically different). Table S3. Comparisons of ligstroside aglycon means (mg/kg) among harvest months using Duncan’s multiple range test (*a* = 0.05) (harvest months reside in the same subset are not statistically different). Table S4. Comparisons of total tyrosol derivatives means (mg/kg) among harvest months using Duncan’s multiple range test (*a* = 0.05) (harvest months reside in the same subset are not statistically different). Table S5. Comparisons of oleacein means (mg/kg) among harvest months using Duncan’s multiple range test (*a* = 0.05) (harvest months reside in the same subset are not statistically different). Table S6. Comparisons of oleuropein aglycon means (mg/kg) among harvest months using Duncan’s multiple range test (*a* = 0.05) (harvest months reside in the same subset are not statistically different). Table S7. Comparisons of dialdehyde ligstroside aglycon means (mg/kg) among harvest months using Duncan’s multiple range test (*a* = 0.05) (harvest months reside in the same subset are not statistically different). Table S8. Comparisons of dialdehyde oleuropein aglycon means (mg/kg) among harvest months using Duncan’s multiple range test (*a* = 0.05) (harvest months reside in the same subset are not statistically different). Table S9. Comparisons of total hydroxy tyrosol derivatives means (mg/kg) among harvest months using Duncan’s multiple range test (*a* = 0.05) (harvest months reside in the same subset are not statistically different). Table S10. Comparisons of variety’s oleacein (mg/kg) means using Duncan’s multiple range test (*a* = 0.05) (varieties reside in the same subset are not statistically different). Table S11. Comparisons of variety’s sum oleocanthal and oleacein (mg/kg) means using Duncan’s multiple range test (*a* = 0.05) (varieties reside in the same subset are not statistically different). Table S12. Comparisons of variety’s oleuropein aglycon (mg/kg) means using Duncan’s multiple range test (*a* = 0.05) (varieties reside in the same subset are not statistically different). Table S13. Comparisons of variety’s ligstroside aglycon (mg/kg) means using Duncan’s multiple range test (*a* = 0.05) (varieties reside in the same subset are not statistically different). Table S14. Comparisons of variety’s dialdehyde ligstroside aglycon (mg/kg) means using Duncan’s multiple range test (*a* = 0.05) (varieties reside in the same subset are not statistically different). Table S15. Comparisons of variety’s dialdehyde oleuropein aglycon (mg/kg) means using Duncan’s multiple range test (*a* = 0.05) (varieties under reside in the same subset are not statistically different). Table S16. Comparisons of variety’s total hydroxy tyrosol derivatives (mg/kg) means using Duncan’s multiple range test (*a* = 0.05) (varieties reside in the same subset are not statistically different). Table S17. Comparisons of variety’s total tyrosol derivatives (mg/kg) means using Duncan’s multiple range test (*a* = 0.05) (varieties under reside in the same subset are not statistically different). Table S18. The distribution of the analyzed olive oil samples according to Greek Region. Table S19. The distribution of the analyzed olive oil samples according to Greek County. Table S20. The distribution of the analyzed olive oil samples according to oil mill type. Table S21. Total phenolic content loss during storage. Figures S1–S8. The distribution of the analyzed olive oil samples according to phenol studied substance. Figure S9. Difference in concentration of oleocanthal, oleacein as well as their sum in relation to harvest month. Figure S10. Difference in concentration of oleuropein aglycon, ligstroside aglycon, dialdehyde ligstroside aglycon and dialdehyde oleuropein aglycon in relation to harvest month. Figure S11. Difference in concentration of total tyrosol and total hydroxy tyrosol derivatives as in total phenols in relation to harvest month.

## Sample Availability

Samples of the compounds are not available from the authors.
