# Supplementary material for: A New Definition of the Term “High-Phenolic Olive Oil” Based on Large Scale Statistical Data of Greek Olive Oils Analyzed by qNMR
Source: Molecules. 2021 Feb 19;26(4):1115. doi: 10.3390/molecules26041115 (PMC7923275; doi:10.3390/molecules26041115)
Supplement: Supplementary file 1 [file molecules-26-01115-s001.pdf]

molecules

MDPI

Article

A new definition of the term "high-phenolic olive oil" based on large scale statistical data of Greek olive oils analyzed by qNMR

Panagiotis Diamantakos[1], Kostas Ioannidis[2], Christos Papanikolaou[1], Annia Tsolakou[1], Aimilia Rigakou[1], Eleni Melliou[1], Prokopios Magiatis[1,*]

[1] Department of Pharmacognosy and Natural Products Chemistry, Faculty of Pharmacy, National and Kapodistrian University of Athens, Panepistimiopolis Zografou, Athens 15771, Greece; pdiam@pharm.uoa.gr (P.D.); papanik@pharm.uoa.gr (C.P.); atsolakou@pharm.uoa.gr (A.T.); aimrigakou@pharm.uoa.gr (A.R); emelliou@pharm.uoa.gr (E.M.); magiatis@pharm.uoa.gr (P.M.)

[2] Laboratory of Sylviculture, Forest Genetics and Biotechnology, Institute of Mediterranean and Forest Ecosystems, Hellenic Agricultural Organization "Demeter", Ilissia, 11528 Athens, Greece; ioko@fria.gr (K.I.)

*Correspondence. magiatis@pharm.uoa.gr; Tel.: +30 210 7274052 (P.M).

Received: date; Accepted: date; Published: date

Table S1. Percentiles (2%) of phenolic content of the examined samples (n=5,764).

Percentiles (%)	Oleocanthal	Oleacein	Sum Oleocanthal Oleacein	Oleuropein Aglycon	Ligstroside Aglycon	Dialdehyde Ligstroside Aglycon	Dialdehyde Oleuropein Aglycon	Total Tyrosol Derivatives	Total Hydroxy Tyrosol Derivatives	Total Phenols
2	0.00	0.00	0.00	0.00	0.00	0.00	0.00	0.00	0.00	0.00
4	28.01	0.00	32.95	0.00	0.00	0.00	0.00	34.60	0.00	40.40
6	38.29	0.00	49.43	0.00	0.00	0.00	0.00	48.46	0.00	65.62
8	44.84	0.00	62.61	0.00	0.00	0.00	0.00	64.26	13.61	88.98
10	51.46	7.97	74.15	0.00	0.00	0.00	0.00	75.79	24.35	110.66
12	56.02	24.14	83.66	0.00	0.00	0.00	0.00	86.19	34.77	128.66
14	60.96	27.93	91.69	9.44	8.95	0.00	0.00	97.99	43.95	149.30
16	65.91	31.35	99.46	11.88	11.28	0.00	0.00	108.75	51.07	167.61
18	69.20	33.06	107.28	14.31	13.61	0.00	0.00	117.37	57.47	185.50
20	74.15	36.48	114.17	14.31	13.61	0.00	0.00	127.20	64.12	198.73
22	77.44	38.19	120.38	16.75	15.94	0.00	0.00	135.14	70.05	213.24
24	80.74	41.61	125.73	16.75	15.94	0.00	0.00	143.50	75.68	227.03
26	85.68	43.32	131.55	19.18	18.26	0.00	0.00	150.73	80.52	239.08
28	88.98	45.14	138.04	19.18	18.26	0.00	0.00	158.66	86.12	251.67
30	92.27	48.44	144.10	20.59	19.18	13.61	0.00	165.20	91.55	263.31
32	97.21	50.15	149.38	21.62	20.59	25.24	0.00	172.61	97.58	274.87
34	100.51	51.86	155.67	22.92	20.59	29.90	0.00	180.08	104.03	288.96
36	103.81	55.28	162.07	24.05	22.92	32.22	0.00	186.30	109.36	302.31
38	107.12	56.99	167.57	24.05	22.92	39.21	9.44	195.01	114.39	316.10
40	110.40	58.70	173.82	26.49	22.92	41.53	14.31	202.92	119.91	329.06
42	113.69	62.12	180.19	26.49	25.24	46.19	16.75	210.79	125.84	342.51

44	117.58	63.82	185.94	28.92	25.24	50.84	21.62	217.83	131.61	356.51
46	121.93	67.24	192.22	28.92	26.49	53.17	24.05	226.18	139.18	367.79
48	125.23	68.95	199.06	29.91	27.57	60.15	26.49	234.53	145.38	383.87
50	130.17	72.37	205.90	31.36	27.57	60.15	28.92	243.13	151.35	399.82
52	133.46	75.79	213.06	32.22	29.90	67.13	33.79	252.12	157.53	413.77
54	138.41	79.21	219.88	33.79	29.90	69.46	33.79	261.45	164.81	429.20
56	143.35	82.62	227.19	34.55	31.36	74.11	38.66	270.53	171.59	446.10
58	148.29	84.35	235.02	36.23	32.22	78.76	43.53	280.25	178.93	461.38
60	151.59	87.75	243.30	36.88	33.79	85.75	43.53	288.95	186.84	479.99
62	158.03	91.17	250.73	38.66	34.55	88.07	48.40	298.87	194.77	498.61
64	164.77	96.30	259.60	41.10	36.23	95.05	53.27	308.98	202.55	515.14
66	168.95	99.72	269.58	41.53	36.88	99.71	55.71	318.73	210.91	535.12
68	174.66	104.84	280.59	43.53	38.66	106.69	60.58	330.37	219.35	554.83
70	181.25	108.26	290.30	45.97	39.30	116.00	63.01	342.70	229.41	575.82
72	187.84	115.10	303.27	47.25	41.53	120.65	70.32	356.80	239.42	600.43
74	196.08	118.52	314.90	48.51	43.86	127.63	72.75	371.51	251.31	625.01
76	204.31	125.35	328.98	50.84	46.19	134.61	80.06	384.19	262.94	647.49
78	214.20	132.19	343.17	53.27	48.51	143.92	84.93	400.39	274.18	671.89
80	222.44	139.03	360.01	57.36	50.84	153.23	92.23	416.90	286.40	700.32
82	233.97	147.57	377.91	60.58	53.17	162.54	101.97	435.37	300.89	734.20
84	247.16	157.83	401.32	63.01	55.49	176.50	111.71	459.26	318.09	768.96
86	257.04	166.37	426.34	67.88	60.12	190.46	121.45	486.69	337.88	815.67
88	273.52	178.33	453.85	72.75	64.79	209.08	136.06	514.92	361.25	870.06
90	291.64	197.65	486.43	80.06	69.46	227.69	153.11	547.42	391.19	927.10
92	320.96	215.93	521.68	87.36	76.44	262.60	175.02	584.36	430.17	1,011.62
94	349.31	238.15	575.60	99.54	85.75	302.16	201.81	638.06	486.10	1,114.53
96	388.86	263.80	641.31	121.45	102.03	348.70	248.07	711.21	558.01	1,235.88
98	457.48	315.06	761.90	177.46	133.72	434.80	309.19	848.79	678.18	1,477.22

Table S2. Comparisons of sum oleocanthal oleacein means (mg/Kg) among harvest months using Duncan's multiple range test (a=0.05) (harvest months reside in the same subset are not statistically different).

**Harvest Month**	**n**	**Subset**			
		**1**	**2**	**3**	**4**
September	163	392.98			
October	948		330.79		
November	1,327			271.35	
December	922				213.83
January	80				187.06

Table S3. Comparisons of ligstroside aglycon means (mg/Kg) among harvest months using Duncan's multiple range test (a=0.05) (harvest months reside in the same subset are not statistically different).

**Harvest Month**	**n**	***Subset***	
		**1**	**2**
September	163	39.88	
November	1,327	37.32	37.32
October	948	36.84	36.84

January	80	34.06	34.06
December	922		32.79

Table S4. Comparisons of total tyrosol derivatives means (mg/Kg) among harvest months using Duncan's multiple range test (a=0.05) (harvest months reside in the same subset are not statistically different).

Harvest Month	n	*Subset*			
		1	2	3	4
September	163	427.68			
October	948		363.97		
November	1,327			307.57	
December	922				244.03
January	80				209.78

Table S5. Comparisons of oleacein means (mg/Kg) among harvest months using Duncan's multiple range test (a=0.05) (harvest months reside in the same subset are not statistically different).

Harvest Month	n	*Subset*		
		1	2	3
October	948	119.94		
September	163	119.74		
November	1,327		103.33	
December	922			80.72
January	80			69.93

Table S6. Comparisons of oleuropein aglycon means (mg/Kg) among harvest months using Duncan's multiple range test (a=0.05) (harvest months reside in the same subset are not statistically different).

Harvest Month	n	*Subset*	
		1	2
October	948	44.31	
November	1,327	43.98	
September	163	41.94	
December	922		33.72
January	80		32.76

Table S7. Comparisons of dialdehyde ligstroside aglycon means (mg/Kg) among harvest months using Duncan's multiple range test (a=0.05) (harvest months reside in the same subset are not statistically different).

Harvest Month	n	*Subset*	
		1	2
October	948	116.28	
September	163	114.56	
November	1,327	102.23	
December	922		78.12
January	80		58.58

Table S8. Comparisons of dialdehyde oleuropein aglycon means (mg/Kg) among harvest months using Duncan's multiple range test (a=0.05) (harvest months reside in the same subset are not statistically different).

Harvest Month	n	*Subset*		
		**1**	**2**	**3**
October	948	67.60		
November	1,327	60.64	60.64	
September	163	56.22	56.22	
December	922		46.04	46.04
January	80			32.45

Table S9. Comparisons of total hydroxy tyrosol derivatives means (mg/Kg) among harvest months using Duncan's multiple range test (a=0.05) (harvest months reside in the same subset are not statistically different).

**Harvest Month**	**n**	*Subset*	
		**1**	**2**
October	948	231.85	
September	163	217.90	
November	1,327	207.95	
December	922		160.48
January	80		135.14

Table S10. Comparisons of variety's oleacein (mg/Kg) means using Duncan's multiple range test (a=0.05) (varieties reside in the same subset are not statistically different).

**Variety**	**n**	**Subset**					
		**1**	**2**	**3**	**4**	**5**	**6**
Lianolia Kerkyras	350	201.74					
Zakynthou	44		163.30				
Olympia	260			137.48			
Kalamon	59			127.45			
Athinolia	260				97.81		
Chalkidikis	362				97.34		
Kolovi	40				96.37		
Agrielia	164				89.79	89.79	
Koroneiki	2,649				81.25	81.25	
Koutsourelia	189				80.02	80.02	
Amfissas	276				79.59	79.59	
Manaki	261					71.34	
Megaritiki	81						49.64

Table S11. Comparisons of variety's sum oleocanthal and oleacein (mg/Kg) means using Duncan's multiple range test (a=0.05) (varieties reside in the same subset are not statistically different).

**Variety**	**n**	**Subset**						
		**1**	**2**	**3**	**4**	**5**	**6**	**7**
Kalamon	59	610.10						

Lianolia Kerkyras	350		499.72					
Zakynthou	44			432.93				
Olympia	260				339.51			
Chalkidikis	362					280.80		
Agrielia	164					276.30		
Athinolia	260					248.13	248.13	
Amfissas	276						227.89	
Koutsourelia	189						227.73	
Kolovi	40						219.58	
Koroneiki	2,649						218.04	
Manaki	261							173.45
Megaritiki	81							148.03

Table S12. Comparisons of variety's oleuropein aglycon (mg/Kg) means using Duncan's multiple range test (a=0.05) (varieties reside in the same subset are not statistically different).

Variety	n	Subset					
		1	2	3	4	5	6
Olympia	260	117.53					
Zakynthou	44		69.71				
Athinolia	260			48.47			
Chalkidikis	362			40.92	40.92		
Koroneiki	2,649				38.16		
Lianolia Kerkyras	350				36.42		
Amfissas	276				36.06		
Agrielia	164				31.74	31.74	
Kalamon	59				31.30	31.30	
Kolovi	40				30.59	30.59	
Koutsourelia	189				30.49	30.49	
Megaritiki	81					23.92	23.92
Manaki	261						18.14

Table S13. Comparisons of variety's ligstroside aglycon (mg/Kg) means using Duncan's multiple range test (a=0.05) (varieties reside in the same subset are not statistically different).

Variety	n	Subset						
		1	2	3	4	5	6	7
Olympia	260	98.51						
Zakynthou	44		57.45					
Athinolia	260			40.46				
Chalkidikis	362			36.23	36.23			
Koroneiki	2,649			33.20	33.20			
Lianolia Kerkyras	350			32.80	32.80			
Agrielia	164			32.21	32.21			
Kalamon	59				31.39	31.39		

Amfissas	276				30.57	30.57		
Koutsourelia	189				28.17	28.17	28.17	
Kolovi	40					22.81	22.81	22.81
Megaritiki	81						21.49	21.49
Manaki	261							17.49

Table S14. Comparisons of variety's dialdehyde ligstroside aglycon (mg/Kg) means using Duncan's multiple range test (a=0.05) (varieties reside in the same subset are not statistically different).

Variety	n	Subset						
		1	2	3	4	5	6	7
Olympia	260	260.71						
Zakynthou	44		196.54					
Athinolia	260			154.16				
Koroneiki	2,649				90.20			
Chalkidikis	362				85.53	85.53		
Lianolia Kerkyras	350				84.24	84.24		
Amfissas	276				77.38	77.38	77.38	
Kalamon	59				71.92	71.92	71.92	
Agrielia	164				63.99	63.99	63.99	
Kolovi	40				60.32	60.32	60.32	
Megaritiki	81					58.50	58.50	
Koutsourelia	189						52.90	52.90
Manaki	261							30.26

Table S15. Comparisons of variety's dialdehyde oleuropein aglycon (mg/Kg) means using Duncan's multiple range test (a=0.05) (varieties under reside in the same subset are not statistically different).

Variety	n	Subset					
		1	2	3	4	5	6
Olympia	260	170.19					
Zakynthou	44		123.43				
Athinolia	260			93.97			
Koroneiki	2,649				54.72		
Lianolia Kerkyras	350				51.76		
Kolovi	40				45.05	45.05	
Amfissas	276				42.79	42.79	
Chalkidikis	362				42.67	42.67	
Agrielia	164				39.79	39.79	39.79
Megaritiki	81				33.09	33.09	33.09
Koutsourelia	189					25.55	25.55
Kalamon	59					25.17	25.17
Manaki	261						20.22

Table S16. Comparisons of variety's total hydroxy tyrosol derivatives (mg/Kg) means using Duncan's multiple range test (a=0.05) (varieties reside in the same subset are not statistically different).

Variety	*n*	Subset						
		1	2	3	4	5	6	8
Olympia	260	425.20						
Zakynthou	44		356.44					
Lianolia Kerkyras	350			289.92				
Athinolia	260				240.25			
Kalamon	59					183.91		
Chalkidikis	362					180.93		
Koroneiki	2,649					174.14	174.14	
Kolovi	40					172.01	172.01	
Agrielia	164					161.31	161.31	
Amfissas	276					158.44	158.44	
Koutsourelia	189						136.06	136.06
Manaki	261							109.71
Megaritiki	81							106.65

Table S17. Comparisons of variety's total tyrosol derivatives (mg/Kg) means using Duncan's multiple range test (a=0.05) (varieties under reside in the same subset are not statistically different).

Variety	*n*	Subset								
		1	2	3	4	5	6	7	8	9
Kalamon	59	585.97								
Olympia	260	561.25	561.25							
Zakynthou	44		523.62							
Lianolia Kerkyras	350			415.02						
Athinolia	260				344.94					
Chalkidikis	362				305.23	305.23				
Agrielia	164					282.71				
Koroneiki	2,649					260.18	260.18			
Amfissas	276					256.25	256.25			
Koutsourelia	189						228.78	228.78		
Kolovi	40							206.34	206.34	
Megaritiki	81								178.38	178.38
Manaki	261									149.86

Table S18. The distribution of the analyzed olive oil samples according to Greek Region.

Region	Frequency	Percent	Cumulative Percent
No Data/mixed origin	740	12.8	12.8
Eastern Macedonia and Thrace	86	1.5	14.3
Attica	125	2.2	16.5
North Aegean	58	1.0	17.5

Crete	525	9.1	26.6
Western Greece	386	6.7	33.3
Western Macedonia	33	.6	33.9
Ionian Islands	811	14.1	48.0
Epirus	65	1.1	49.1
Central Macedonia	353	6.1	55.2
South Aegean	81	1.4	56.6
Peloponnese	2,294	39.8	96.4
Central Greece	82	1.4	97.8
Thessaly	125	2.2	100.0
**Total**	5,764	100.0	

Table S19. The distribution of the analyzed olive oil samples according to Greek County.

County	Frequency	Percent	Cumulative Percent
No Data/mixed origin	788	13.7	13.7
Achaia	98	1.7	15.4
Aetolia and Akarnania	108	1.9	17.2
Argolida	308	5.3	22.6
Arkadia	182	3.2	25.7
Attica	125	2.2	27.9
Boeotia	28	.5	28.4
Chalkidiki	310	5.4	33.8
Chania	215	3.7	37.5
Chios	7	0.1	37.6
Cyclades	60	1.0	38.7
Dodecanesos	16	0.3	38.9
Drama	3	0.1	39.0
Evia	26	0.5	39.5
Evros	4	0.1	39.5
Fokida	5	0.1	39.6
Ilia	179	3.1	42.7
Irakleio	114	2.0	44.7
Kavala	58	1.0	45.7
Kefalonia	109	1.9	47.6
Kerkyra	355	6.2	53.7
Korinthos	194	3.4	57.1
Kozani	33	0.6	57.7
Lakonia	614	10.7	68.3
Larisa	7	0.1	68.5
Lasithi	159	2.8	71.2
Lefkada	60	1.0	72.3
Lesvos	48	0.8	73.1
Magnessia	89	1.5	74.6
Messinia	994	17.2	91.9
Phthiotida	23	0.4	92.3
Preveza	58	1.0	93.3
Rethymno	32	0.6	93.8

Rodopi	2	0.0	93.9
Samos	3	0.1	93.9
Thesprotia	7	0.1	94.0
Thessaloniki	21	0.4	94.4
Trikala	14	0.2	94.7
Xanthi	19	0.3	95.0
Zakynthos	289	5.0	100.0
**Total**	5,764	100.0	

Table S20. The distribution of the analyzed olive oil samples according to oil mill type.

Oil Mill Type	Frequency	Percent	Cumulative Percent
No data	2,833	49.1	49.1
Two-Phase	1,880	32.6	81.8
Three-Phase	1,051	18.2	100.0
Total	5,764	100.0	

Table S21. Total phenols preservation (%) during three different long-term storage treatments.

Storage treatment	Time (months)		
	3	6	12
Room Temperature	87.72% ± 6.61%	78.10% ± 9.70%	54.10% ± 9.46%
4°C	-	92.06% ± 7.35%	84.18% ± 5.28%
-18°C	-	96.47% ± 2.65%	90.50% ± 3.08%

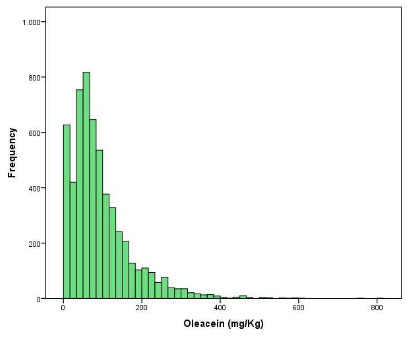

S1

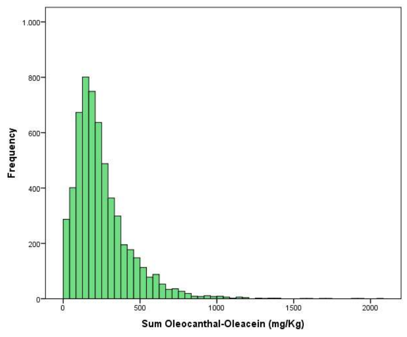

S2

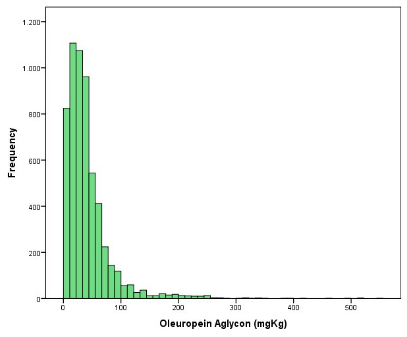

S3

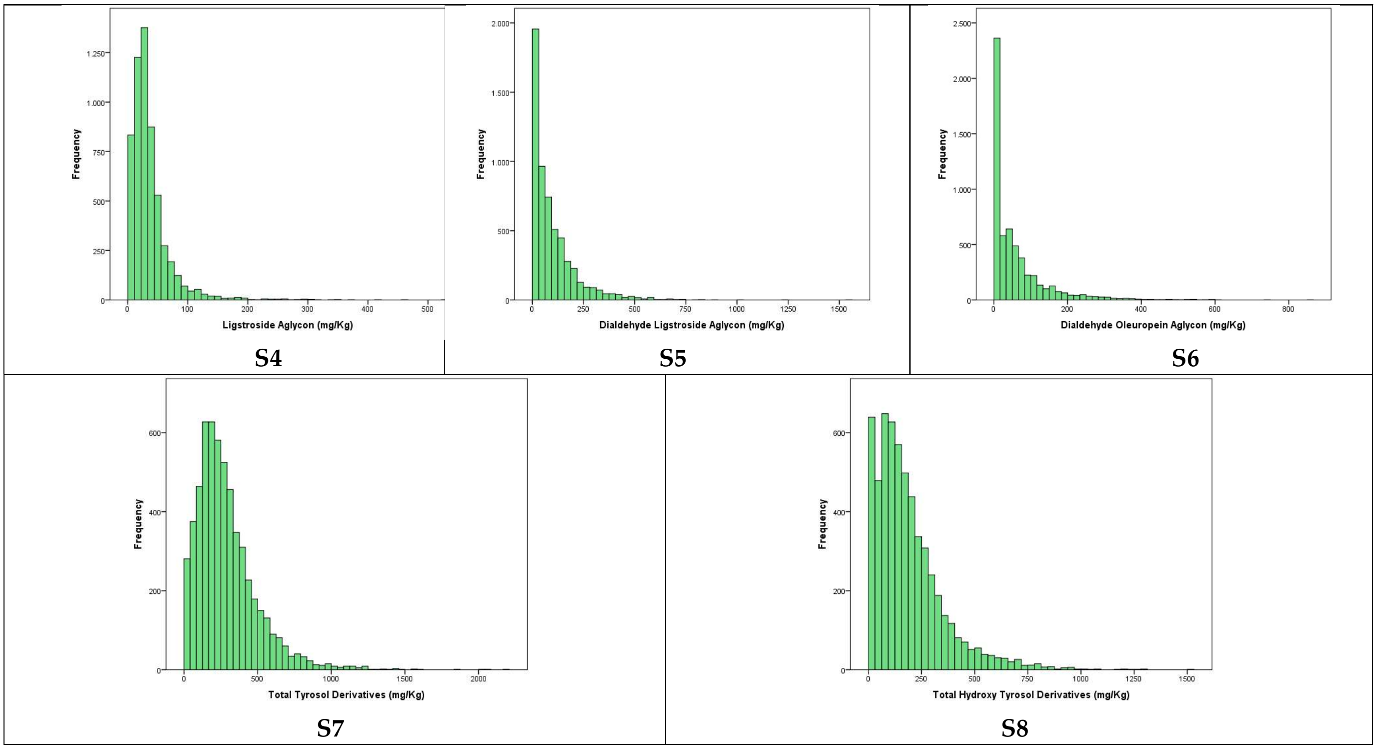

Figure S1-S8. The distribution of the analyzed olive oil samples according to phenol studied substance.

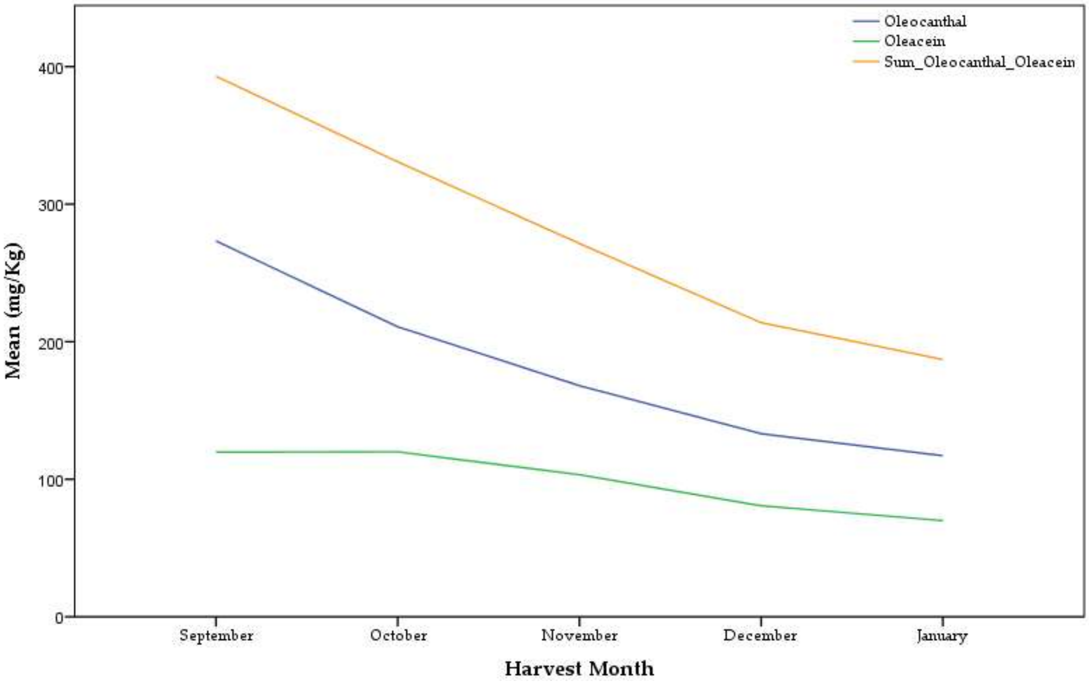

Figure S9. Difference in concentration of oleocanthal, oleacein as well as their sum in relation to harvest month.

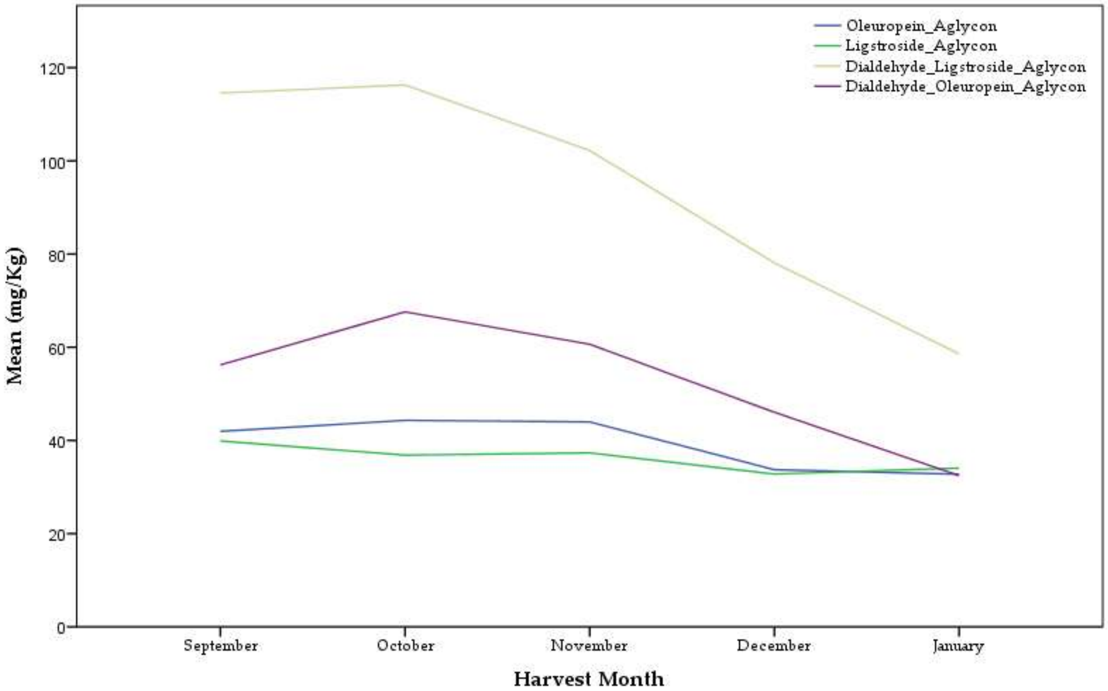

Figure S10. Difference in concentration of oleuropein aglycon, ligstroside aglycon, dialdehyde ligstroside aglycon and dialdehyde oleuropein aglycon in relation to harvest month.

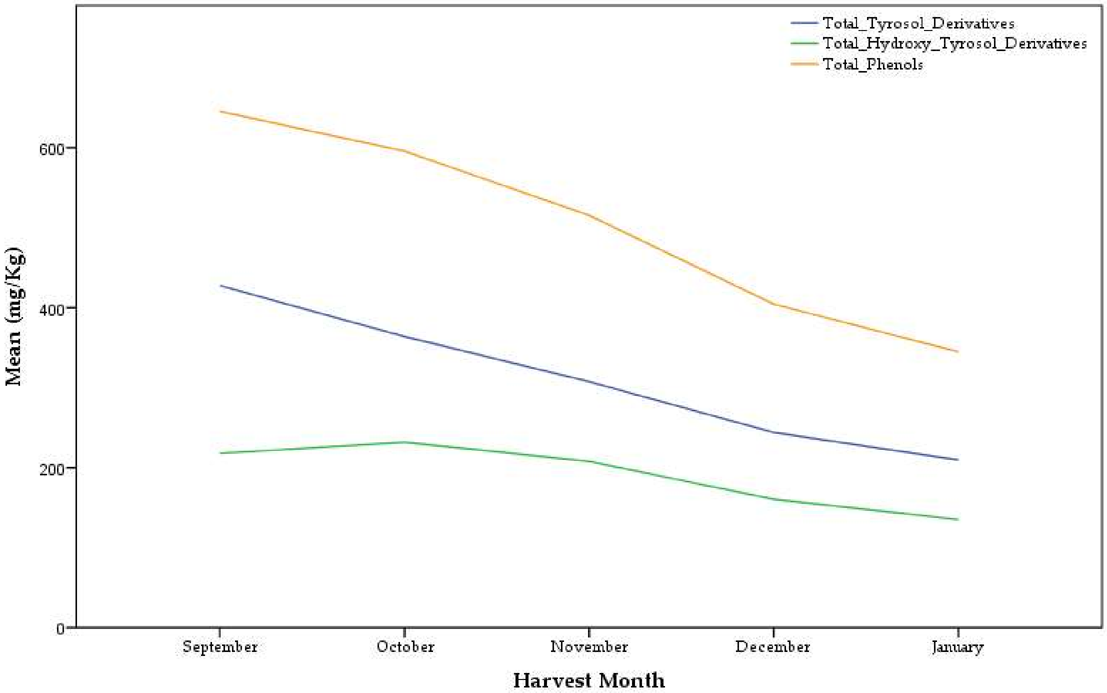

Figure S11. Difference in concentration of total tyrosol and total hydroxy tyrosol derivatives as in total phenols in relation to harvest month.
